# Systematic review and meta-analysis comparing ventilatory support in chemical, biological and radiological emergencies*

**DOI:** 10.1590/1518-8345.4024.3347

**Published:** 2020-08-31

**Authors:** Israel Baptista de Souza Borges, Magali Rezende de Carvalho, Marcel de Souza Quintana, Alexandre Barbosa de Oliveira

**Affiliations:** 1 Universidade Federal do Rio de Janeiro, Escola de Enfermagem Anna Nery, Rio de Janeiro, RJ, Brazil.; 2 Universidade Federal Fluminense, Escola de Enfermagem Aurora Afonso Costa, Niterói, RJ, Brazil.; 3 Fundação Oswaldo Cruz, Instituto Nacional de Infectologia, Rio de Janeiro, RJ, Brazil.

**Keywords:** Airway Management, Personal Protective Equipment, Intubation, Laryngeal Masks, Meta-Analysis, Disasters, Manuseio das Vias Aéreas, Equipamento de Proteção Individual, Intubação, Máscaras Laríngeas, Metanálise, Desastres, Manejo de la Vía Aérea, Equipo de Protección Personal, Intubación, Máscaras Laríngeas, Metaanálisis, Desastres

## Abstract

**Objective:**

to compare the mean development time of the techniques of direct laryngoscopy and insertion of supraglottic devices; and to evaluate the success rate in the first attempt of these techniques, considering health professionals wearing specific personal protective equipment (waterproof overalls; gloves; boots; eye protection; mask).

**Method:**

meta-analysis with studies from LILACS, MEDLINE, CINAHL, Cochrane, Scopus and Web of Science. The keywords were the following: *personal protective equipment; airway management; intubation; laryngeal masks*.

**Results:**

in the “reduction of the time of the procedures” outcome, the general analysis of the supraglottic devices in comparison with the orotracheal tube initially presented high heterogeneity of the data (I^2^= 97%). Subgroup analysis had an impact on reducing heterogeneity among the data. The “laryngeal mask as a guide for orotracheal intubation” subgroup showed moderate heterogeneity (I^2^= 74%). The “2^nd^generation supraglottic devices” subgroup showed homogeneity (I^2^= 0%). All the meta-analyses favored supraglottic devices. In the “success in the first attempt” outcome, moderate homogeneity was found (I^2^= 52%), showing a higher proportion of correct answers for supraglottic devices.

**Conclusion:**

in the context of chemical, biological or radiological disaster, the insertion of the supraglottic device proved to be faster and more likely to be successful by health professionals. PROSPERO record (CRD42019136139).

## Abstract (pt)

**Objetivo:**

comparar o tempo médio do desenvolvimento das técnicas de laringoscopia direta e de inserção de dispositivos supraglóticos e avaliar a taxa de sucesso na primeira tentativa dessas técnicas, considerando a utilização de equipamentos de proteção individual específicos (macacão impermeável; luvas; botas; proteção ocular; máscara) pelos profissionais de saúde.

**Método:**

metanálise com estudos das bases LILACS, MEDLINE, CINAHL, Cochrane, Scopus e Web of Science. As palavras-chave foram: *personal protective equipment; airway management; intubation; laryngeal masks*.

**Resultados:**

no desfecho redução “do tempo dos procedimentos” a análise geral dos dispositivos supraglóticos em comparação com o tubo orotraqueal apresentou inicialmente alta heterogeneidade dos dados (I^2^ = 97%). A análise por subgrupos impactou na redução da heterogeneidade entre os dados. O subgrupo “máscara laríngea como guia para intubação orotraqueal” demonstrou heterogeneidade moderada (I^2^ = 74%). O subgrupo “dispositivos supraglóticos de 2ª geração” evidenciou homogeneidade (I^2^ = 0%). Todas as metanálises foram favoráveis aos dispositivos supraglóticos. No desfecho “êxito na primeira tentativa” foi encontrada homogeneidade moderada (I^2^ = 52%), demonstrando maior proporção de acerto para dispositivos supraglóticos.

**Conclusão:**

no contexto do desastre químico, biológico ou radiológico, a inserção do dispositivo supraglótico revelou ser mais rápida e apresentar maior chance de acerto por profissionais de saúde. Registro PROSPERO (CRD42019136139).

## Abstract (es)

**Objetivo:**

comparar el tiempo medio de desarrollo de las técnicas de laringoscopia directa y de inserción de dispositivos supraglóticos y evaluar la tasa de éxito obtenida en el primer intento de dichas técnicas con profesionales sanitarios provistos de equipos de protección individual específicos (mono impermeable, guantes, botas, protección ocular, mascarilla).

**Método:**

meta-análisis con estudio de las bases de datos LILACS, MEDLINE, CINAHL, Cochrane, Scopus y Web of Science. Las palabras clave fueron las siguientes: *personal protective equipment; airway management; intubation; laryngeal masks*.

**Resultados:**

en el resultado “reducción del tiempo de los procedimientos”, el análisis general de los dispositivos supraglóticos en comparación con el tubo orotraqueal presentó, inicialmente, una alta heterogeneidad de los datos (I^2^= 97%). El análisis por subgrupos dio como resultado una reducción de la heterogeneidad entre los datos. El subgrupo “mascarilla laríngea como guía para la intubación orotraqueal” mostró una heterogeneidad moderada (I^2^= 74%). El subgrupo “dispositivos supraglóticos de 2ª generación” mostró homogeneidad (I^2^= 0%). Todos los meta-análisis fueron favorables a los dispositivos supraglóticos. En el resultado “éxito en el primer intento” se halló una homogeneidad moderada (I^2^= 52%), obteniendo una mayor proporción de aciertos con el uso de dispositivos supraglóticos.

**Conclusión:**

en el contexto de un desastre químico, biológico o radiológico, se ha demostrado que la inserción del dispositivo supraglótico es más rápida y que los profesionales sanitarios obtienen una mayor probabilidad de acierto. Registro PROSPERO (CRD42019136139).

## Introduction

Several emergency situations are triggered by the malicious or accidental use of chemical, biological or radiological agents, which can result in respiratory failure for the victims. The terrorist attacks in Syria with the Sarin neurotoxic gas in 2013 and 2017 can be cited^(^ 1 ^)^. In Brazil, in 2013, in the city of Santa Maria - Rio Grande do Sul, there was an accidental fire in a nightclub, which generated the emission of hydrogen cyanide, a chemical agent that, at that time, was responsible for the increase in clinical cases of acute respiratory failure, which resulted in the drastic death of 242 people and respiratory distress in approximately 1,000 victims^(^ 2 ^)^.

As for the biological agents, in West Africa, the Ebola virus epidemic in 2014 triggered an international civic-military response that left several countries on alert. The epidemic claimed the lives of thousands^(^ 3 ^-^ 4 ^)^. In 2015, in Brazil, the first suspected case of Ebola was reported, which mobilized the health sector and provided the opportunity for the preparation of health professionals in relation to the specific protective equipment they should wear, as well as transportation and adequate health care addressed to the person with the suspected condition. This initiative was justified by the pathophysiology of the disease itself, which stimulates an inflammatory response, followed by an immuno-suppressive phase, causing respiratory failure due to blood aspiration and septic shock^(^ 5 ^-^ 6 ^)^.

In relation to the inadvertent use of radiological agents, in 1987, in Goiânia, a municipality belonging to the state of Goiás (Brazil), a relevant radiological accident with cesium-137 had worldwide repercussion from the rupture of a lead capsule of an abandoned radiotherapy device in a disabled clinic. The consequence was the monitoring of 112,000 citizens, health care for 249 irradiated or contaminated people and four deaths, in addition to the marks it left as the worst radiological disaster in the world that occurred outside nuclear plants^(^ 7 ^)^.

Faced with such situations, when considering that first aids often occur with the victim still contaminated by a chemical, biological or radiological agent, it is recommended that at least the health professional use level C personal protective equipment, which essentially consists of waterproof overalls, gloves, boots, eye protection, and mask^(^ 3 ^,^ 8 ^-^ 10 ^)^.

In view of the dimension and severity that events of this nature can cause, it is necessary to provide adequate protection to the health professionals, especially to the nursing team, who must be able to fully exercise their craft to alleviate suffering and care for the people affected in a safe and fruitful way. Indeed, the procedures analyzed and the individual protection equipment indicated to the health professionals in these complex circumstances confirm the need to discuss better standards of response and risk management, as well as the importance of having effective health technologies in calamitous scenarios^(^ 11 ^)^.

Additionally, it is worth mentioning the authorization issued by the Federal Nursing Council (Brazil), through its Opinion No. 1/2015, for trained nurses to proceed with the insertion of supraglottic devices, in case of urgencies and emergencies. In this sense, in a scenario of emergencies and disasters involving chemical, biological or radiological agents, the autonomy of these professionals is reasserted in the management of the airways in assisting victims with respiratory failure^(^ 12 ^)^.

In face of the agents that cause respiratory failure, there are procedures, such as direct laryngoscopy and the insertion of supraglottic devices, for establishing a patent airway and, consequently, a reduced risk of death^(^ 13 ^-^ 14 ^)^.

However, there are divergences among studies on the “reduction of time to perform the procedure” and “success in the first attempt of each applied technique” outcomes. As an example, there is a study that recommends direct laryngoscopy^(^ 15 ^)^, as well as a study recommending supraglottic devices^(^ 16 ^)^, both published in 2018. For this reason, a meta-analysis was deemed necessary, since these outcomes are crucial as they directly impact the survival of victims of respiratory failure in a calamitous scenario, and provide substantial subsidies in the decision-making of the health professional about which technology should be used as a priority.

Therefore, the objectives of this study are to compare the mean of development time of the techniques of direct laryngoscopy and insertion of supraglottic devices; and to evaluate the success rate in the first attempt of these techniques, considering health professionals wearing specific personal protective equipment (waterproof overalls; gloves; boots; eye protection; mask).

## Method

This systematic review and meta-analysis, under PROSPERO (International Prospective Register of Ongoing Systematic Reviews) record CRD42019136139, followed the guidelines recommended by the Preferred Reporting Items for Systematic Reviews and Meta-Analyses (PRISMA). The MEDLINE (1946-2019), Cochrane (1999-2019), Scopus (1966-2019), Web of Science (1900-2019), LILACS (1987-2019), and CINAHL (1961-2019) databases were used, in addition to a manual search in the reference list of the selected studies, a search in the list of studies related to each eligible study on the PubMed platform, and a search in Google Scholar. The inclusion criteria adopted were the following: randomized clinical trial with comparison between orotracheal intubation and insertion of supraglottic devices performed by health professionals using level C personal protective equipment. The exclusion criteria were the following: randomized clinical trial with comparison between orotracheal intubation and insertion of supraglottic devices performed by health professionals using level C personal protective equipment on children or pediatric-sized mannequins; and studies that did not provide the necessary data for the meta-analysis, such as number of participants, mean time and standard deviation. The study collection period was between March 8^th^ and August 30^th^, 2019; and there were no restrictions as for language or year of publication.

The search strategies were configured as follows:

In MEDLINE: (((((((respiratory insufficiency[MeSH Terms]) OR manikins[MeSH Terms]) OR nerve agents[MeSH Terms]) OR chemical warfare agents[MeSH Terms]) OR hazardous substances[MeSH Terms])) AND (((((airway management[MeSH Terms]) OR intubation, Intratracheal[MeSH Terms]) OR Personal Protective Equipment[MeSH Terms]) OR protective clothing[MeSH Terms]) OR laryngoscopy[MeSH Terms])) AND laryngeal masks[MeSH Terms].

In Scopus: ( TITLE-ABS-KEY ( airway AND management ) OR TITLE-ABS-KEY ( manikin* ) AND TITLE-ABS-KEY ( personal AND protective AND equipment ) OR TITLE-ABS-KEY ( cbrn ) OR TITLE-ABS-KEY ( cbrn AND ppe ) OR TITLE-ABS-KEY ( chemical AND suit ) OR TITLE-ABS-KEY ( protective AND clothing ) AND TITLE-ABS-KEY ( intubation ) ).

In Cochrane: (airway*): ti,ab,kw AND (intubation*):ti,ab,kw AND (“personal protective equipment”*):ti,ab,kw OR (protective clothing*):ti,ab,kw AND (laryngeal mask*):ti,ab,kw. Below is the strategy in Web of Science: ALL FIELDS:(airway management) AND TITLE:(manikin*) OR TITLE: (cadaver*) AND TITLE: (personal protective equipment) OR TITLE: (CBRN-PPE) AND ALL FIELDS: (intubation) AND ALL FIELDS: (laryngeal mask*) OR ALL FIELDS: (supraglottic). Refined by: WEB OF SCIENCE CATEGORIES: (EMERGENCY MEDICINE).

The studies derived from the application of the strategies were selected by two independent reviewers and filtered by reading their titles and abstracts, with due registration on the studies’ eligibility form. After checking the lists, a Kappa coefficient of 0.74 was obtained, a satisfactory value that reflected the objectivity and clarity of the data to be collected^(^ 17 ^)^. Disagreements regarding the inclusion of studies were resolved in a consensus meeting. Subsequently, citations were exported to the EndNote online reference manager. All the studies from this first selection were analyzed in their full texts.

The data collected from each study were organized in a data extraction instrument containing the following: authors’ names; title; country of origin; year and magazine of publication; study population and environment; types of ventilatory support devices; number of participants; mean time spent performing the technique; standard deviation; and success rate in the first attempt of each procedure.

According to the Cochrane collaboration tool, for assessing the risk of bias in randomized studies, each eligible study was classified as low, moderate, or high risk of bias^(^ 18 ^)^.

The meta-analysis was developed using the RevMan 5.3 software, whose data were worked under random effect, with calculation of the difference in time means for the first objective, and the difference of proportion/risk for the second objective. When the study did not present all the data for meta-analysis, but offered a means for calculating it, the data was calculated. The I test was considered low heterogeneity with I^2^ < 50%; moderate heterogeneity with I^2^ test between 50 and 75%; and high heterogeneity with I^2^ test > 75%^(^ 19 ^)^. Additionally, the analysis was treated by subgroups when I^2^> 50%. The significance level was set at 0.05. The GRADE system was used to assess the quality of the evidence, classified as high, moderate, low, and very low, as well as the strength of the recommendation, classified as strong or weak^(^ 20 ^)^.

## Results

**Figure 1:**
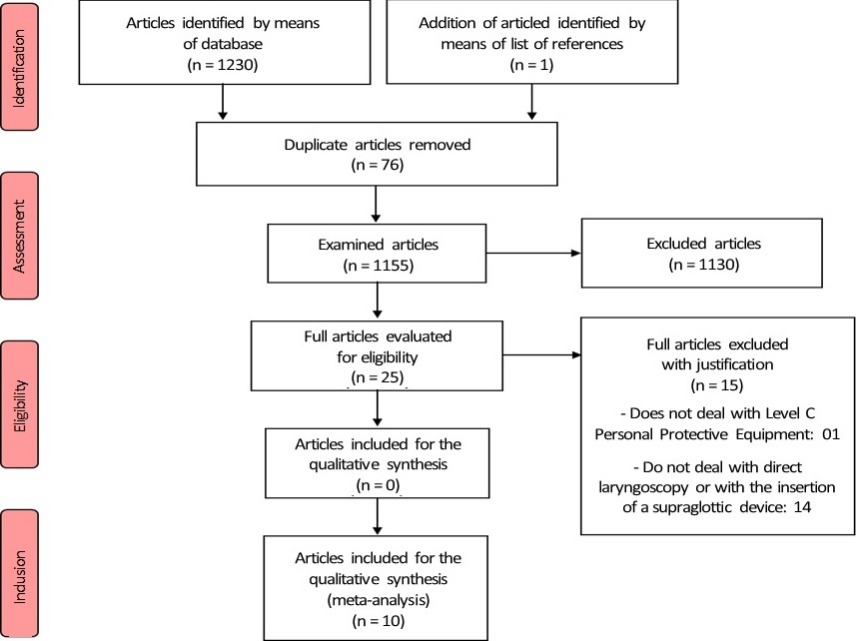
Flowchart of the Systematic Review in PRISMA format

Figure 1 shows the product of the search strategies used.

In the sequence, Figure 2 represents the characterization of the 10 included studies, where all are prospective and of the crossover type; that is, the participants experienced the application of techniques with the supraglottic device and the orotracheal tube.

**Figure 2:** Characterization of the studies

Authors (Year)	Assessed technology	Participants (n)	Number of attempts	Technique application instrument
Ben-Abraham; Weinbroum (2004)^(^ 21 ^)^	1^st^generation laryngeal mask; Orotracheal tube	Non-anesthetist health professional (10); Anesthetist (10)	2	Adult size Laerdal Airway Management Trainer mannequin
Castle, et al. (2009)^(^ 22 ^)^	1^st^generation laryngeal mask; Orotracheal tube	Prehospital physician (4); Resuscitation officer (6); Paramedic (14), Anesthetist (15); Emergency physician (25).	2	Adult size Laerdal Airway Management Trainer mannequin
Castle, et al. (2010)^(^ 23 ^)^	1^st^generation laryngeal mask; Orotracheal tube	Pre-hospital care physician; Resuscitation officer; Paramedic; Senior and junior anesthetist; Senior and junior emergency physician (75).	2	Adult size Laerdal Airway Management Trainer mannequin
Flaishon, et al. (2004)^(^ 24 ^)^	1^st^generation laryngeal mask; Orotracheal tube	Anesthetist (15)	1	Human being
Garner; Laurence; Lee (2004)^(^ 10 ^)^	1^st^generation laryngeal mask; Orotracheal tube	Paramedic (3); Emergency physician (3); Anesthetist (2)	1	Adult mannequin
Greenland, et al. (2007)^(^ 25 ^)^	ILMA* laryngeal mask; Orotracheal tube	Senior and Junior Anesthetist (18)	1	Adult size Laerdal Airway Management Trainer mannequin
Ophir, et al.(2014)^(^ 26 ^)^	1^st^generation laryngeal mask; 2^nd^generation laryngeal mask; Laryngeal tube; Orotracheal tube	Anesthetist (20); Rescuer (26), Paramedic (27), General practitioner (24); Medical resident of different specialties (20)	3	Adult size Laerdal Airway Management Trainer mannequin
Plazikowski, et al. (2018)^(^ 16 ^)^	i-gel laryngeal mask; ILMA* laryngeal mask Orotracheal tube	Physician qualified as an anesthetist and emergency physician (30)	1	Adult size Laerdal Airway Management Trainer mannequin
Wang, et al.(2016)^(^ 27 ^)^	1^st^generation laryngeal mask; Orotracheal tube	Senior and junior emergency physician (40)	1	Adult size Laerdal Airway Management Trainer mannequin
Weaver, et al. (2015)^(^ 28 ^)^	ILMA* laryngeal mask; Orotracheal tube	Junior Emergency Physician (37)	3	Adult size Laerdal Airway Management Trainer mannequin

* ILMA = Laryngeal mask capable of guiding orotracheal intubation

In the selected studies, a comparison between 1^st^ and 2^nd^generation supraglottic devices was identified, comprising models of laryngeal mask and laryngeal tube. This classification is based on the chronological evolution and improvement of intrinsic characteristics of the supraglottic devices such as the angle of curvature for better accommodation in the larynx; the sealing ability in the larynx to withstand pressure ventilation; a composition that is difficult to deform in case of bites by the victim; and the creation of a gastric drainage route^(^ 13 ^)^.

It should be noted that there were studies that compared more than one supraglottic device with the orotracheal tube, which made meta-analyses and their recommendations more robust.

It is worth mentioning that there were two studies whose lead author was an English nurse^(^ 22 ^-^ 23 ^)^, with 139 participants performing 278 times with the supraglottic device, and 258 times with the orotracheal tube. This represents 33.61% of the supraglottic device data and 31.97% of the orotracheal tube data for the meta-analyses. Such information reiterates the importance of this theme for nursing care practices in emergencies and disasters.

**Figure 3:**
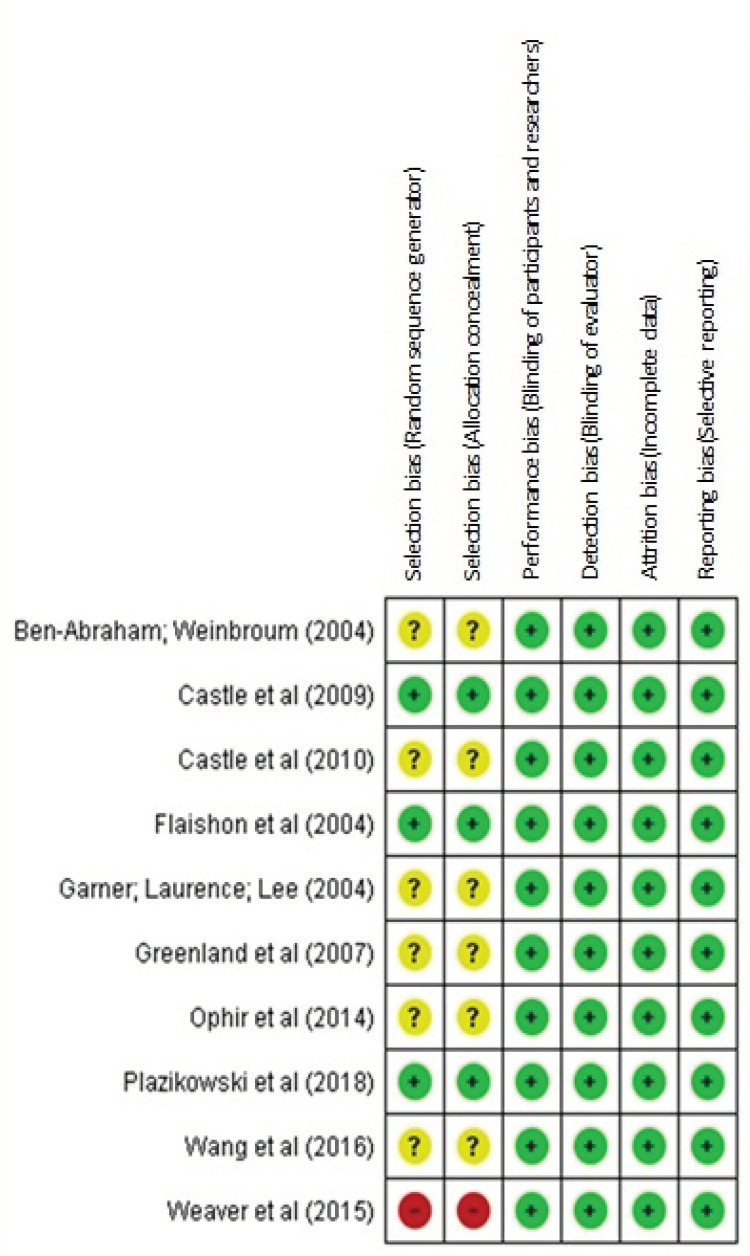
Bias risk assessment of the studies

Using the Cochrane collaboration tool, the quality of each selected study was assessed. As shown in Figure 3, the following domains were evaluated: selection bias, performance bias, detection bias, attrition bias, and reporting bias^(^ 18 ^)^.

When considering that the final classification of the study should be based on the highest risk of bias found among the domains, selection bias stood out as the main criterion for attributing a moderate risk of bias. Three studies with a low risk of bias, six studies with a moderate risk of bias, and one study with a high risk of bias were identified. It was prioritized that the study reported how the random sequence and allocation secrecy were generated. Low risk of performance and detection bias was attributed to studies due to textual evidence in the sources of information and explanation of the extra author acquired via e-mail. Low risk was attributed in relation to attrition bias, as there was no provision of incomplete data. Regarding reporting bias, a low risk of bias was attributed, since no trace of attempted data manipulation was identified.

**Figure 4:**
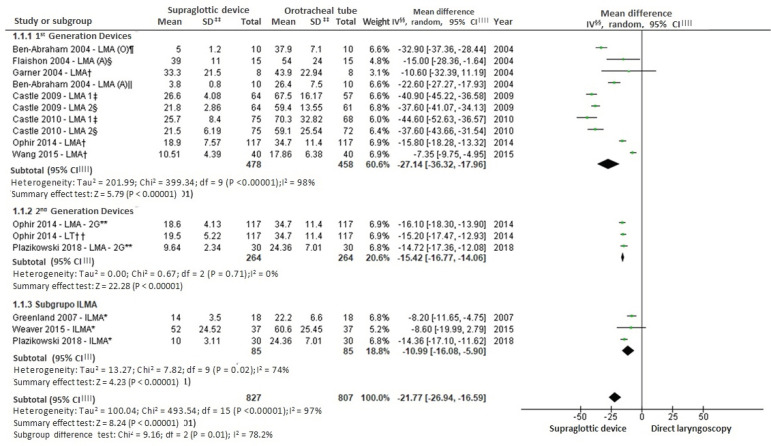
Mean time of the techniques of direct laryngoscopy and insertion of supraglottic devices

**Figure 5:**
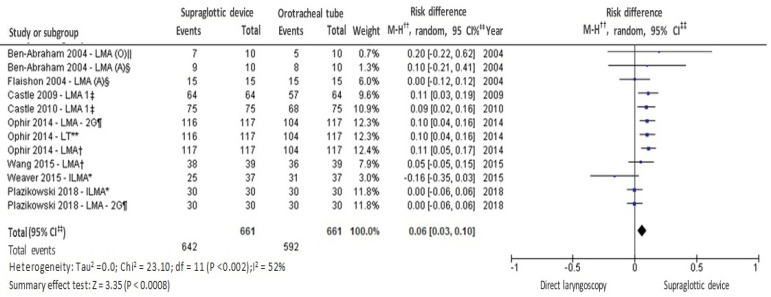
Success rate in the first attempt at orotracheal intubation and insertion of supraglottic device ^*^ILMA = Laryngeal mask capable of guiding orotracheal intubation; ^†^LMA = Laryngeal mask; ^‡^LMA1 = Laryngeal mask 1^st^attempt; ^§^ LMA (A) = Laryngeal mask (anesthesiologists); ^ǁ^LMA (O)=Laryngeal mask (another non-anesthesiologist); ^¶^ LMA-2G = 2^nd^generation laryngeal mask; **LT = Laryngeal tube; ^††^MH = Mantel-Haenszel; ^‡‡^CI = Confidence interval

According to Figure 4, it is observed that the supraglottic insertion technique obtained a lower mean of time spent compared to the direct laryngoscopy technique with the orotracheal tube. The general and subgroup result was statistically significant and favorable to the supraglottic device. Regarding the evaluation of the success rate in the first attempt of each applied technique, and when considering the use of level C personal protective equipment by the health professionals, eight studies were collected for meta-analysis. Moderate heterogeneity of significant effect and statistically significant summary effect were identified. The result was favorable for the supraglottic device, as shown in Figure 5.

## Discussion

For the “reduction of time spent on each procedure” outcome, the systematic review shows that the mean time to insert supraglottic devices is shorter compared to the orotracheal tube. Subgroup analysis was useful, as it reasserted favoring supraglottic devices through statistically significant results.

Concerning the second outcome, “success in the first attempt”, the result showed moderate heterogeneity with significant effect (p = 0.02) and a statistically significant summary effect in the difference of proportion: 0.06 [95% CI: 0.03; 0.10]; (p = 0.0008); I^2^= 52%. In the analysis of the success rate in the first attempt of each applied technique, this systematic review obtained 89.56% for direct laryngoscopy and 97.12% for the insertion of a supraglottic device. The results of the systematic review were reinforced by other similar results, with 85% success rate for direct laryngoscopy^(^ 29 ^)^, and 100% for the supraglottic device^(^ 30 ^)^.

The evaluation of the level of evidence presented in the studies by the GRADE system, with regard to the “reduction of time for performing direct laryngoscopy compared to the insertion of a supraglottic device” outcome, found a moderate level of evidence. Regarding the “success in the first attempt of each technique” outcome, a moderate level of evidence was also obtained. Demonstrated by the GRADE system, a strong recommendation force that favors 2^nd^generation supraglottic devices and the laryngeal masks capable of guiding orotracheal intubation, and a weak recommendation strength was identified in relation to the 1^st^generation supraglottic devices.

In accident scenarios with multiple victims, with or without involvement of a chemical, biological or radiological agent, one of the objectives is to provide a prior airway for as many people as possible^(^ 24 ^,^ 30 ^)^. Therefore, the use of supraglottic devices by the health professionals with personal protective equipment in these scenarios has been systematically studied. In general, the results point to ease of use and rapid insertion^(^ 30 ^-^ 32 ^)^. In addition, less stringent training requirements for its use have been confirmed when compared to orotracheal intubation^(^ 30 ^-^ 31 ^)^.

Another aspect highlighted is related to the clinical conditions of seriously infected victims, who require advanced immediate life support, which includes the use of ventilatory support techniques, a priority to reduce mortality^(^ 33 ^)^. Even in these cases, orotracheal intubation is the gold standard to ensure the airway and prevent death^(^ 34 ^)^.

In the case of the evolution of 2^nd^generation supraglottic devices, feasible improvements have made them more useful and applicable in different environments, like the pre-hospital, in-hospital emergency, and surgical environments^(^ 24 ^)^. There are currently a variety of 2^nd^generation supraglottic devices, which have similar characteristics, such as anti-bite system, route for gastric emptying, better airway sealing, and structure to be a ventilatory support route under high pressures. Such characteristics make supraglottic devices a technology that has been gaining space and importance in the daily care of the health professionals^(^ 13 ^,^ 24 ^,^ 32 ^)^. It can be inferred that the effectiveness and the performance among the 2^nd^generation supraglottic devices of this systematic review reflected in the homogeneity of this subgroup.

In addition, emergency orotracheal intubation outside the operating room is associated with significant operational challenges, which include a higher incidence of difficult intubation due to environmental factors, possible lack of experience in laryngoscopy, rapid deterioration of clinical conditions, and risk of regurgitation^(^ 13 ^)^.

Paradoxically, despite all the selected studies, a study was retrieved, whose participants were emergency physicians with level C personal protective equipment, performing orotracheal intubation and the insertion of supraglottic devices in cadavers^(^ 15 ^)^. The results suggest orotracheal intubation as a faster technique, with statistically significant data (p = 0.02) and with a higher success rate in the first attempt (direct laryngoscopy = 58% and supraglottic device = 21%)^(^ 15 ^)^. However, it is noteworthy that this study was eliminated from this systematic review because it did not present data with the respective standard deviation or means for calculating it, which made insertion in the meta-analysis unfeasible, in compliance with the provisions of the inclusion criteria. Thus, a high risk of attrition bias was identified due to the fact that it did not present the standard deviation in the study. It is worth mentioning that few participants in this study had previous experience with a supraglottic device, which meant that the researchers promoted a preliminary training, in which each participant performed the two procedures three times. These same participants had an Advanced Life Support Course, and reported having performed direct laryngoscopy at least 30 times in their career. In view of the above, after assessing the risk of bias by the highest degree identified in the domains, the general assessment of this study^(^ 15 ^)^ was considered high risk, weakening the reliability of its results. This was the only study that was contrary to the favorable trend towards supraglottic devices.

Regarding the potential limitations of this study, it is emphasized that the systematic review evaluated different types of supraglottic devices, as well as it considered different methodologies, professional categories or areas of activity, which reflected in heterogeneity. However, the summary effect was strongly favorable to supraglottic devices, in all the meta-analyses.

Another limitation concerns the instruments on which the ventilatory support techniques were applied. As explained in Figure 2 on the characterization of the eligible studies, mannequins and adult humans were used. The selection brought together nine studies with mannequins, and one study with 60 human beings submitted to surgical and orthopedic interventions. The decision to jointly analyze both studies on mannequins and human beings maintained an adequate comparative uniformity, as both were immobile and without reaction during the interventions. In addition, the patients were relatively healthy; sedated and relaxed; monitored and with venous access; in optimal hemodynamic and ventilatory conditions; no reactions during the insertion of devices^(^ 24 ^)^. The inclusion of this study with humans in the meta-analyses reinforced the trend of studies on mannequins. However, it must be admitted that if all the studies involved human beings, which was not found, the impact and applicability of the recommendations generated by this systematic review would be greater. In addition, the study participants treated one patient or one mannequin at a time; did not see oral cavity with secretions; nor were there conditions for multiple injuries, seizures or hypoxia. Such description does not correspond to a real intervention on a victim in frank respiratory insufficiency by a chemical, biological or radiological agent, in the historical context of an accident with multiple victims, in which the objective is to provide a safe airway for the largest number of victims possible with chances to survive. Finally, the time until the airway is installed is a crucial factor for survival and stabilization of the victim who is physically compromised, unconscious, dyspnoic, with increased secretions in the airways and hypoxemic^(^ 10 ^,^ 35 ^)^.

Therefore, it should be considered that nine of the ten studies in this systematic review were carried out in a skills laboratory, that is, in a closed environment, with ideal brightness, controlled temperature and mannequins, which may not reflect reality^(^ 10 ^,^ 14 ^,^ 35 ^)^. In addition to the above, scholars in the field comment on the difficulty of developing research studies of this nature with human beings, in relation to the use of chemical warfare agents^(^ 14 ^)^.

## Conclusion

The systematic review points to supraglottic devices as being faster to the detriment of orotracheal intubation, under the “reduction in the time to perform ventilatory support techniques” outcome. Thus, with a moderate level of evidence, supraglottic devices are recommended as the first choice in an emergency and disaster scenario involving chemical, biological or radiological agents, which may involve the need to care for multiple contaminated victims, where road management aerial is required. Subsequently, once the patient is stabilized, the environment is better known and has a greater supply of professionals and material, the supraglottic device can be exchanged for the orotracheal tube.

Regarding the “success in the first attempt” outcome there is a moderate level of evidence that the supraglottic device has a higher percentage of correct responses compared to the orotracheal tube.

These outcomes are important because they directly impact the survival of a victim in respiratory failure and provide substantial subsidies in the decision-making process of the health professionals, nurse included, on which technology should be used as a priority because it is faster and more likely to be successful on the first attempt.

The GRADE system found a strong recommendation force, which favored the indication of 2nd generation supraglottic devices and of the laryngeal masks capable of guiding orotracheal intubation; and a weak recommendation strength was identified favoring 1st generation supraglottic devices.

Despite the advances and recommendations that this systematic review has provided, it is still necessary to develop clinical studies with better methodological robustness, in order to obtain recommendations with a higher level of evidence, especially due to the need for safety and quality of care.
